# Identification of ONECUT3 as a stemness-related transcription factor regulating NK cell-mediated immune evasion in pancreatic cancer

**DOI:** 10.1038/s41598-023-45560-y

**Published:** 2023-10-24

**Authors:** Haojun Shi, Yiusing Tsang, Yisi Yang, Hok Leong Chin

**Affiliations:** 1 Department of General Surgery, Ruijin Hospital, Shanghai Jiao Tong University School of Medicine, Shanghai, China; 2 Shanghai Institute for Endocrine and Metabolic Diseases, Ruijin Hospital, Shanghai Jiao Tong University School of Medicine, Shanghai, China; 3 Graduate School of Asia-Pacific Studies, Waseda University, Tokyo, Japan; 4 Department of Pediatrics, The University of Chicago, Chicago, IL USA

**Keywords:** Pancreatic cancer, Cancer microenvironment, Cancer stem cells, Tumour immunology, Tumour heterogeneity, Immune evasion, Bioinformatics

## Abstract

Pancreatic ductal adenocarcinoma (PDAC) has a dismal response to the current T cell-based immunotherapies, which is attributed to intratumoral heterogeneity caused by PDAC stem cells and lack of major histocompatibility complex class I required for neoantigen presentation. Although this scenario makes natural killer (NK) cells attractive candidates for immunotherapeutic agents targeting MHC-I-deficient cancer stem cells in heterogeneous PDACs, little is known about PDAC stem cell immunology. In our study, PDAC-specific datasets from public databases were collected for in-depth bioinformatic analysis. We found that the abundance of PDAC stemness negatively influenced the infiltration of NK cells and identified the transcription factor ONECUT3 enriched in PDACs with high stemness index scores and Pan-cancer Stemness Signature levels. A series of NK cell-targeted inhibitory immune checkpoints were highly expressed in ONECUT3^high^ PDACs. The patient group with high levels of ONECUT3 expression had a high risk of poor overall survival, even if accompanied by high infiltration of NK cells. Furthermore, the prostanoid metabolic process was enriched in ONECUT3^high^ PDACs with high levels of NK cell-targeted inhibitory immune checkpoints. ONECUT3 enriched in high-stemness PDACs possessed the potential to transcriptionally regulate the prostanoid metabolism-related genes. Our study reveals ONECUT3 as a candidate stemness-related transcription factor regulating NK cell-targeted inhibitory immune checkpoints in PDAC. ONECUT3-mediated prostanoid metabolism may regulate cancer stemness and immune evasion in PDAC. Synergistic inhibition of prostanoid metabolism may improve the efficacy of NK cell-based immunotherapies targeting intratumoral heterogeneity caused by PDAC stem cells.

## Introduction

Pancreatic ductal adenocarcinoma (PDAC) is projected to become the second leading cause of cancer-related death by 2030 due to a continued increase in incidence and minimal improvement in prognosis^1^. Radical resection and adjuvant chemotherapy can only benefit a minority of patients with resectable PDAC^2^, while locally advanced and metastatic PDACs are refractory to most cytotoxic polychemotherapy^3,4^. In recent years, combining immunotherapy with conventional anti-tumour treatment has marked a therapeutic renaissance in oncology. However, except for less than 1% of microsatellite instability-high tumours^5^, most PDACs have a dismal response to FDA-approved immunotherapies, especially immune checkpoint blockade (ICB)^6^, which show efficacy in some solid tumours including melanoma and non-small cell lung cancer^7,8^. Early strategies of PD-1 inhibitors with gemcitabine and nab-paclitaxel did not exhibit as satisfactory activity as expected in patients with advanced PDAC^9,10^. Dual checkpoint blockade using anti-PD-1 and CTLA-4 antibodies to target non-redundant pathways of T cell inhibition only achieved an overall response rate of 3% in patients with metastatic PDAC^11^. These disappointing results inevitably highlight the limitations of current ICB strategies.

The nearly universal refractoriness of PDAC to T cell-based immunotherapies has been attributed to a low mutation burden predicted to generate very few immunogenic antigens and a complex immunosuppressive tumour microenvironment devoid of CD8^+^ T cells and their activation marker expression^12^. Recently, ample evidence demonstrates that intratumoral heterogeneity–the presence of multiple subclones in cancer progression–hinders clinical responses to immunotherapy^13,14^. Cancer stem cells, as the source of hierarchically organized cancer cell subpopulations, are believed to drive initiation, progression and therapeutic resistance including immunotherapy^14,15^. Inhibiting inhibitory signals from immune checkpoints on cancer stem cells diminished tumour growth and lymph node metastases in preclinical models^16^. Elimination of adaptive immune resistance emerging from cancer stem cells enhanced the tumoricidal activity of adoptive cell therapy^17^. It is possible that precision immunotherapy targeting the immune privilege of pancreatic cancer stemness may improve the efficiency of preventing and treating tumour relapse and distant metastasis. Furthermore, PDAC promotes immune evasion and immunotherapy resistance by repressing the major histocompatibility complex class I (MHC-I) expression^18–20^ indispensable for presenting peptide antigens processed in tumour cells to cytotoxic T cells^21^. Intriguingly, natural killer (NK) cells can recognize and attack abnormal cells that downregulate MHC-I expression to evade T cell responses, and upregulate activating ligands induced by DNA damage or malignant transformation^22^. The human leukocyte antigen (HLA)-unrestricted manner of NK cells makes them attractive candidates for immunotherapeutic agents targeting MHC-I-deficient cancer stem cells in heterogeneous PDAC. However, it is unknown whether PDAC stem cells exert an immunosuppressive effect on the NK cells in the tumour microenvironment, and, if so, whether this can be exploited for the development of NK cell-based precision immunotherapies.

Here, we used the datasets of PDAC from The Cancer Genome Atlas (TCGA), The International Cancer Genome Consortium (ICGC), Tumor Immune Single-cell Hub 2 (TISCH2), The Human Protein Atlas (HPA), the Cancer Therapeutics Response Portal (CTRP) and The Genomics of Drug Sensitivity in Cancer (GDSC) to evaluate the influence of PDAC stemness on the immune microenvironment and identify one stemness-related transcription factor regulating NK cell-targeted inhibitory immune checkpoints in PDAC. The impact of the interaction between PDAC stemness and NK cells on patient prognosis was also estimated to highlight the immune privilege of cancer stem cells against NK cells within stemness-high PDACs. Furthermore, to eliminate immune evasion led by PDAC stem cells, we explored the regulatory pathways of cancer stemness and immune evasion and screened the potentially sensitive drugs. Our findings will advance in-depth knowledge of cancer stem cell immunology and facilitate the development of efficient NK-based immunotherapies targeting PDAC stem cells to prevent and treat cancer recurrence and metastasis.

## Methods

### Study cohort and data collection

RNA sequencing data of PDAC samples and corresponding clinical parameters of patients were derived from TCGA (n = 179) and ICGC (n = 189). RNA sequencing data of normal tissues were derived from TCGA (n = 4) and The Genotype-Tissue Expression (GTEx) (n = 167). Single-cell RNA sequencing data of PDAC and normal pancreas was obtained from TISCH2^23^ and HPA^24^, respectively. Gene expression data and drug activity for PDAC cell lines were obtained from CTRP and GDSC. ONECUT3-based functional protein association networks were obtained from BioGRID^25^ and STRING databases. R version 4.2.3 was used to analyse the data. Because all data that our study used were from publicly available datasets, no ethical approval was required to seek.

### Cancer stemness and immune infiltrate analysis

The stemness index (mRNAsi) was calculated on a one-class logistic regression (OCLR) machine learning algorithm and represented the stemness features of tumour samples at the transcriptomic levels^26^. The closer mRNAsi score was to 1, the more stem-like tumour cells. Meanwhile, a pan-cancer stemness signature derived from machine learning models was used to predict immunotherapy responses influenced by cancer stemness (GSVA package)^27^. The deconvolution algorithm ESTIMATE was utilized to estimate tumour purity, the level of stromal cells, and the infiltration level of immune cells in PDAC tissues (estimate package)^28^. Another three deconvolution algorithms quanTIseq, MCP-counter and single-sample GSEA (ssGSEA) were utilized to quantify the immune contexture in the tumour microenvironment from bulk RNA sequencing data^29–31^. quanTIseq deduced a global picture of stromal cells, including B cells, M1 and M2 macrophages, monocytes, neutrophils, NK cells, CD4^+^ T cells, CD8^+^ T cells, regulatory T cells (Tregs) and myeloid dendritic cells (quantiseqr package). MCP-counter showed the abundance of stromal cells including T cells, CD8^+^ cells, cytotoxic lymphocytes, NK cells, B lineage, monocytic lineage, myeloid dendritic cells, and neutrophils (MCPcounter package). ssGSEA identified the dynamics of 28 different immune cell types infiltrating tumours (GSVA package). In addition, PDACs were classified into five immune subtypes associated with prognosis, genetic, and immune modulatory alterations that may shape the specific types of immune microenvironments (ImmuneSubtypeClassifier package), including wounding healing, interferon-γ (IFN-γ), inflammatory, lymphocyte depleted, transforming growth factor-β (TGF-β)^32^.

### Identification of differentially expressed genes and gene set enrichment analysis

Differentially expressed genes between samples with low and high mRNAsi scores, or ONECUT3 expression, were identified by the DESeq2 package. An adjusted *p*-value < 0.05 and |log2 (Fold Change)|> 1 were used to indicate statistical significance. Gene set enrichment analysis (GSEA) was conducted to figure out the pathways enriched in PDACs with high stemness or ONECUT3 expression to explore the underlying mechanisms (clusterProfiler package). The inclusion criteria were |normalized enrichment score (NES)|> 1, nominal (NOM) *p*-value < 0.05 and FDR < 0.25.

### Comparison, correlation and survival analysis

Normalized gene expression was presented in the form of log2(TPM + 1) when comparison or correlation analysis was performed. Tumour purity adjustment was adopted to reduce the bias caused by stromal cells in the heterogeneous microenvironment. We conducted purity-corrected partial Spearman's correlation analysis to recognize the co-expression pattern of ONECUT3 and inhibitory immune checkpoints or the association between ONECUT3 and immune infiltrates (ggplot2 package). Kaplan–Meier curves were used to show patient survival influenced by ONECUT3 expression and immune infiltrates (survival, survminer and ggplot2 package).

### Ethical approval

Because all data this study used was from publicly available databases TCGA, GTEx, ICGC, TISCH2, HPA, CTRP, GDSC, BioGRID and STRING, no ethical approval was required to seek.

## Results

### Association of cancer stemness with immune infiltrates in PDAC

**Figure 1:**
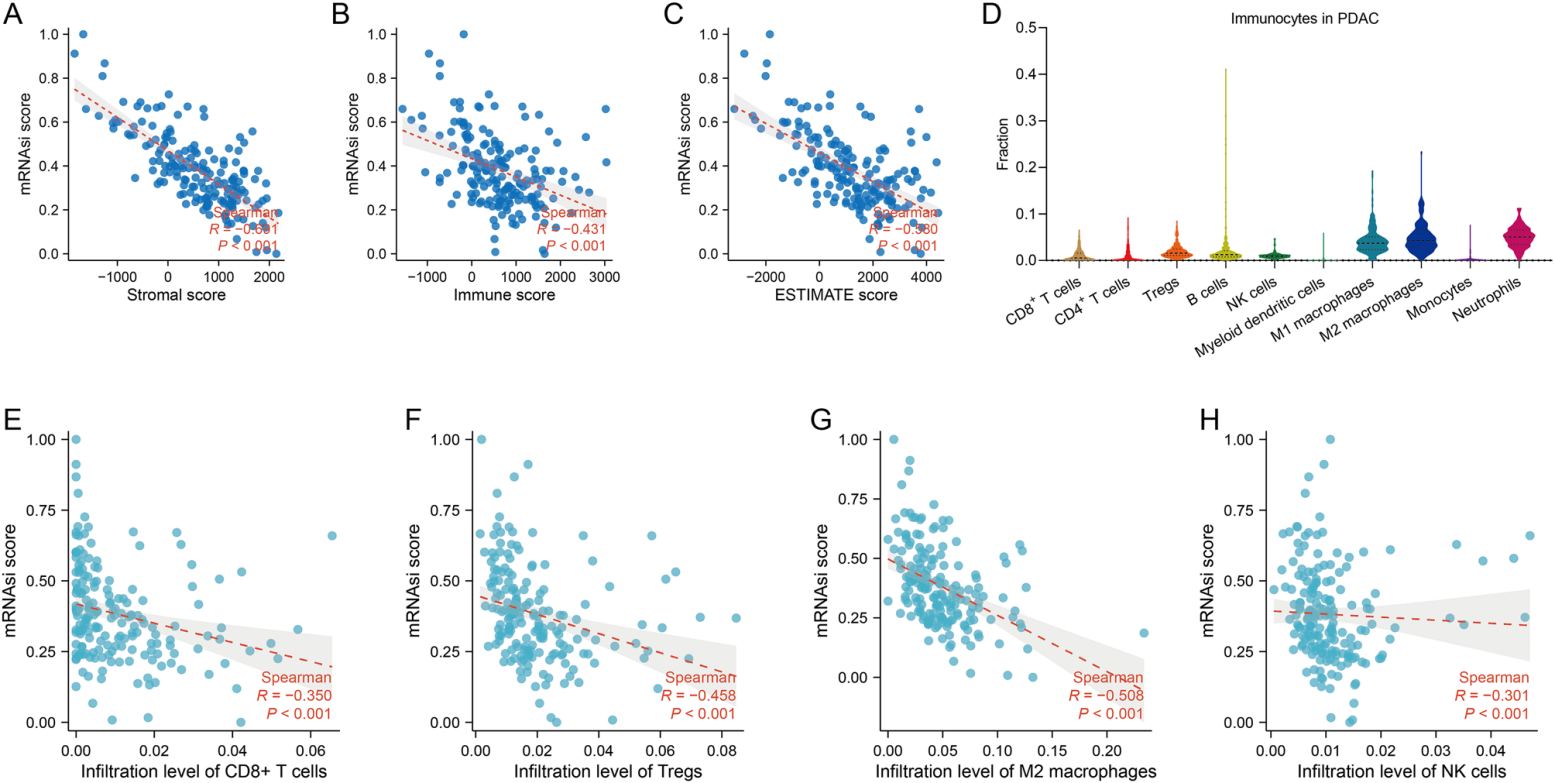
Association of cancer stemness with immune infiltrates in the PDAC patient cohort from TCGA. (**A**–**C**) Spearman’s correlation of mRNAsi scores with (**A**) Stromal scores, (**B**) Immune scores and (**C**) ESTIMATE scores in PDAC (based on ESTIMATE algorithm). (**D**) The landscape of immune infiltrates in PDAC (based on quanTIseq algorithm). (**E**–**N**) Spearman’s correlation of mRNAsi scores with infiltration of (**E**) CD8^+^ T cells, (**F**) Tregs, (**G**) M2 macrophages, (**H**) NK cells in PDAC (based on quanTIseq algorithm).

Cancer stem cells have been proposed to evade immune surveillance in the tumour microenvironment by developing diverse mechanisms^33^. To verify whether cancer stemness influences immune infiltrates in PDAC, we first calculated mRNAsi, an mRNA expression-based stemness index, to evaluate the abundance of cancer stemness, and utilized ESTIMATE, a deconvolution algorithm, to estimate the tumour purity, the level of stromal cells, and the infiltration level of immune cells in PDAC tissues. Correlation analysis showed that the mRNAsi scores of the six gastrointestinal cancer types were all associated with the ESTIMATE scores (Fig. 1C, Supplementary Fig. S4C, Fig. S5C, Fig. S6C, Fig. S7C, Fig. S8C), implying that high cancer stemness contributes to a heterogeneous tumour microenvironment. Meanwhile, the mRNAsi scores of ESCA, PDAC, READ and STAD were significantly associated with the Immune scores (Fig. 1B, Supplementary Fig. S5B, Fig. S7B, Fig. S8B) while no such association was found in COAD and LIHC (Supplementary Fig. S4B, Fig. S6B). The mRNAsi scores of the six gastrointestinal cancer types were also associated with the Stromal scores (Fig. 1A, Supplementary Fig. S4A, Fig. S5A, Fig. S6A, Fig. S7A, Fig. S8A). These results suggest that cancer stemness may hurt the infiltration of immune cells and stromal cells into tumour tissue across six gastrointestinal cancer types.

To depict the landscape of the immune microenvironment, we used quanTIseq, a deconvolution algorithm, to characterize the cell composition from their gene expression profiles. Neutrophils and M1 macrophages were enriched across all six gastrointestinal cancer types, whereas adaptive immune cells, including NK cells, CD8^+^ T cells and B cells, occupied only a limited fraction (Fig. 1D). Furthermore, to analyse which type of immune cell cancer stemness may restrain, we investigate the association of cancer stemness enrichment with the infiltration of different immune cells across six gastrointestinal cancer types. The mRNAsi scores of PDAC were significantly associated with the infiltration of NK cells and CD8^+^ T cells (Fig. 1E,H), while no association of the mRNAsi scores with CD4^+^ T cells, B cells, myeloid dendritic cells, monocytes, M1 macrophages and neutrophils was observed in PDAC (Supplementary Fig. S1). To enhance the credibility, we also utilized other algorithms, such as MCPcounter and ssGSEA, to assess immune cell infiltration. As expected, the association of the mRNAsi scores with the infiltration of NK cells and CD8^+^ T cells was observed in PDAC (Supplementary Fig. S2A and D, Fig. S3G and J). These results demonstrate that the cancer stemness of PDAC may impede the infiltration of the adaptive immune system and that PDACs with high stemness tend to have fewer tumour-infiltrating NK cells and CD8^+^ T cells which can be primed and activated for immunotherapy. Although the negative correlation of cancer stemness with the infiltration of Tregs and M2 macrophages was observed in PDAC (Fig. 1F,G), it cannot be denied that the tumour-promoting effect of Tregs and M2 macrophages persists and that the suppressive effect of cancer stemness on the overall immune microenvironment is not covered up.

### Identification of a stemness‑related transcription factor regulating immune evasion in PDAC

**Figure 2:**
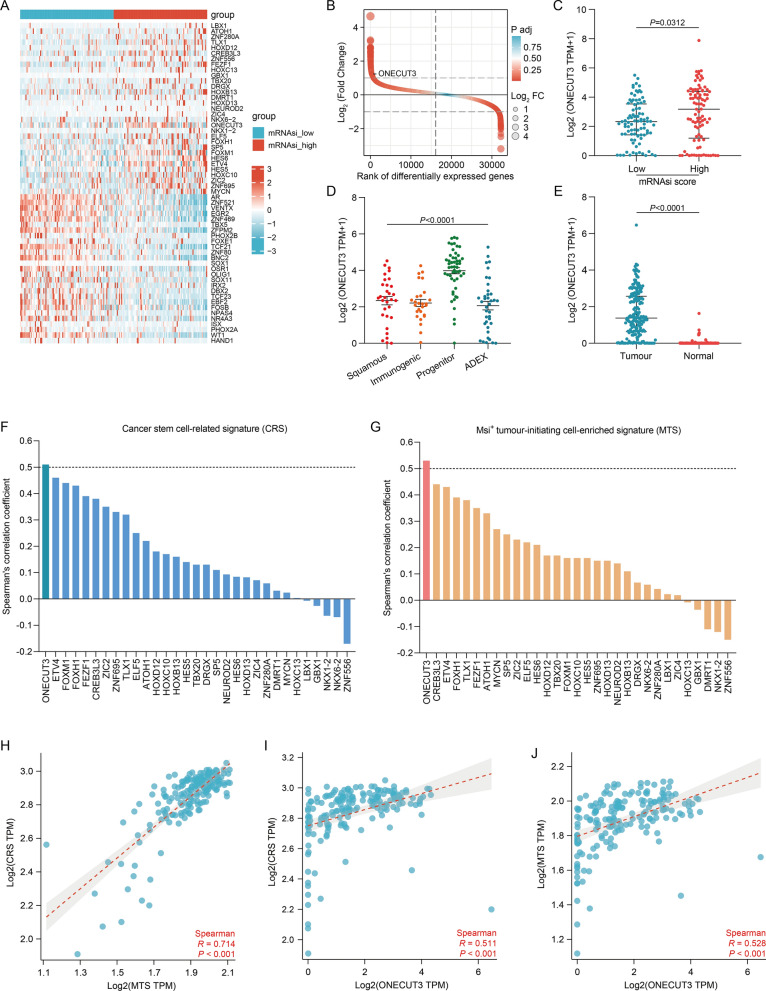
Identification of one stemness-related transcription factor in PDAC. (**A**) Heatmap of differentially expressed transcription factors in PDACs with low and high mRNAsi scores. (**B**) The rank of differentially expressed transcription factors according to log2(Fold Change). (**C**) Differential levels of ONECUT3 expression in PDACs with low and high mRNAsi scores. (**D**) Differential levels of ONECUT3 expression in the four subtypes of PDAC. ADEX, aberrantly differentiated exocrine. (**E**) Differential levels of ONECUT3 expression in PDAC (from TCGA) and normal tissues (from TCGA and GTEx). (**F**) Spearman's correlation of the transcription factors with cancer stem cell-related signature (CRS) in PDAC. The threshold of the correlation coefficient was set at 0.5. (**G**) Spearman's correlation of transcription factors with Msi^+^ tumour-initiating cell-enriched signature (MTS) in pancreatic cancer. The threshold of the correlation coefficient was set at 0.5. (**H**) Spearman's correlation between CRS and MTS in PDAC. (**I**) Spearman's correlation between ONECUT3 expression and CRS in PDAC. (**J**) Spearman's correlation between ONECUT3 expression and MTS in PDAC.

Since transcription factors are the key controllers of intrinsic cellular processes and cellular responses to environmental perturbations^34^, we intended to find the transcription factor that functions in cancer stemness and immune evasion. The differentially expressed genes between PDACs with high and low mRNAsi scores were compared to explore the changes at the transcriptomic level. The selection criteria, adjusted *p*-value < 0.05 and |log2FC|> 1, resulted in the identification of thirty-one up-regulated and twenty-seven down-regulated genes that coded transcription factors (Fig. 2A,B). We also compared the gene expression between high and low levels of Pan-cancer Stemness Signature (PSS) and found five up-regulated and nineteen down-regulated genes that coded transcription factors (Supplementary Fig. S9). We then evaluated the correlation of those up-regulated transcription factor genes with two individual sets of signatures representative of PDAC stem cells. Cancer Stem Cell-Related Signature (CRS) is enriched in cancer stem cell-like ductal cells figured out by single-cell RNA sequencing^35^. Msi^+^ Tumour-Initiating Cell-Enriched Signature (MTS) was constructed by comparing the gene expression profiles of Msi^+^ tumour-initiating cells and differentiated tumour cells^36^. As expected, CRS and MTS were closely correlated in PDAC (Fig. 2H), demonstrating that either of the two individual signatures can forcefully forecast PDAC stemness. Correlation analysis showed that ONECUT3 expression was the most closely correlated with CRS and MTS among the thirty-one up-regulated genes (Fig. 2B,F,G,I,J, Supplementary Fig. S10). Besides, the levels of ONECUT3 expression were significantly higher in the high mRNAsi score group than in the low mRNAsi score group (Fig. 2C). Higher levels of ONECUT3 expression were also observed in the PDAC tissues than in the normal pancreatic tissues (Fig. 2E). Intriguingly, the levels of ONECUT3 expression were the highest in the pancreatic progenitor tumours among the four subtypes of PDAC (Fig. 2D). Since pancreatic progenitor tumours preferentially express genes pivotal for pancreatic endoderm cell-fate determination towards a pancreatic lineage^37^, ONECUT3 may also be responsible for PDAC initiation and indispensable for establishing a whole PDAC cell hierarchy. Furthermore, we observed ONECUT3 expression in PDAC and normal pancreas at single-cell level. Single-cell analysis demonstrated high levels of ONECUT3 expression in malignant cells and low levels of ONECUT3 expression in ductal cells (Supplementary Fig. S11), which is consistent with Fig. 2E. Taken together, ONECUT3 was identified as a potential stemness-related transcription factor in PDAC.

**Figure 3:**
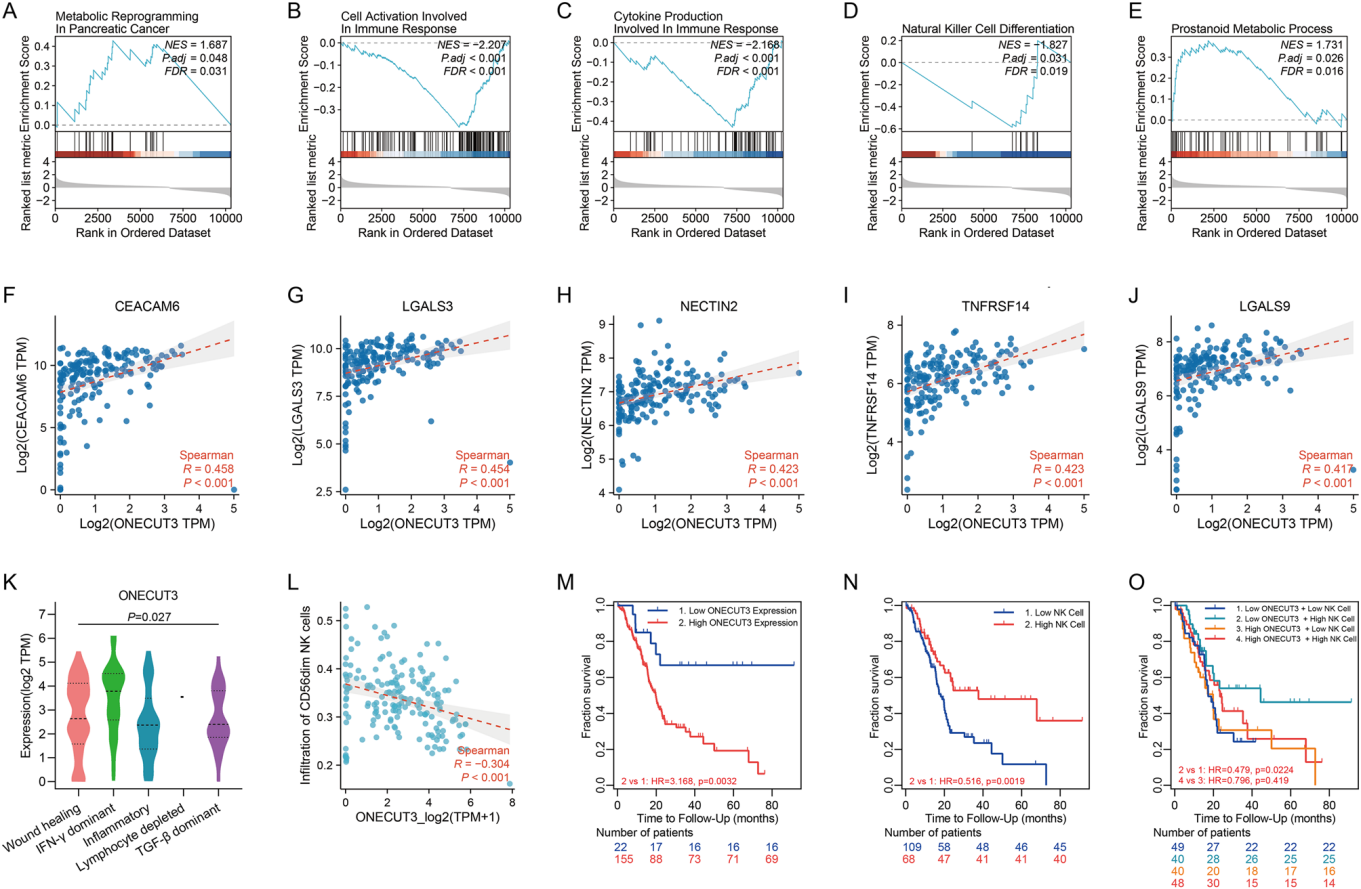
Impact of the stemness-related transcription factor ONECUT3 on tumour-infiltrating NK cells in PDAC. (**A**–**E**) GSEA of differentially expressed genes between PDACs with high and low mRNAsi scores. (**F**–**J**) Spearman's correlation of ONECUT3 expression with (**F**) CEACAM6, (**G**) LGALS3, (**H**) NECTIN2, (**I**) TNFRSF14 and (**J**) LGALS9 expression in PDAC. (**K**) Violin plots for ONECUT3 expression in multiple immune subtypes of PDAC. (**L**) Spearman's correlation between ONECUT3 expression and infiltration of CD56^dim^ NK cells. (**M**) Kaplan–Meier curves for overall survival split by level of ONECUT3 expression in PDAC. (**N**) Kaplan–Meier curves for overall survival split by infiltration level of NK cells in PDAC. (**O**) Kaplan–Meier curves for overall survival split by level of ONECUT3 expression and infiltration level of NK cells in PDAC.

To characterize the pancreatic cancer stemness and figure out the way of immune evasion, we performed GSEA by comparing the high and low mRNAsi score groups of PDACs and found that reprogramming in the prostanoid metabolic process was enriched in PDACs with high stemness, while the transcriptional signature of high PDAC stemness was negatively correlated with cell activation and cytokine production involved in immune response, and NK cell differentiation (Fig. 3A–E). Metabolic reprogramming in PDAC is a feature of pancreatic progenitor tumours^37^ where ONECUT3 was highly expressed (Fig. 2D). Failure of immune activation and NK cell differentiation in PDACs with high stemness hints at the possibility that ONECUT3 may promote immune evasion by weakening the function of NK cells. Given that the tumoricidal activity of NK cells is mediated by a diverse repertoire of inhibitory receptors and their corresponding ligands^22^, we intended to identify inhibitory immune checkpoints regulated by the stemness-related transcription factor ONECUT3. When correlation analysis was performed, tumour purity adjustment was adopted to reduce the bias caused by the mixture with immune cells during data processing. ​The expression levels of CEACAM6, LGALS3, NECTIN2, TNFRSF14 and LGALS9 were found to be closely correlated with those of ONECUT3, respectively (Fig. 3F–J), while no correlation was obtained between the expression levels of other inhibitory immune checkpoints and ONECUT3 (Supplementary Fig. S12A and B). Of note, LGALS3, NECTIN2 and LGALS9 have been identified as inhibitory ligands for NK cell activation^38^. Furthermore, to clarify the clinical significance of the stemness-related transcription factor, we estimated the influence of ONECUT3 on the infiltration and activity of immune cells by correlation and survival analysis. ONECUT3 expression was negatively associated with CD56^dim^ NK cells (Fig. 3L), while it was slightly associated with CD56^bright^ NK cells (Supplementary Fig. S12C). Since CD56^dim^ NK-cell subset is more naturally cytotoxic and CD56^bright^ NK-cell subset has the capacity to produce abundant cytokines including IFN-γ^39^, it appeared reasonable that the levels of ONECUT3 expression were the highest in the IFN-γ dominant PDACs (Fig. 3K) that have the most complex intratumoral heterogeneity with a high proliferation rate^32^. More importantly, we confirmed the negative impact of high ONECUT3 expression levels on the overall survival of PDAC patients (Fig. 3M). The result that the PDAC patient group with high infiltration of NK cells had better overall survival (Fig. 3N) reflected that NK cells did play a role in the clearance of tumour cells. Strikingly, any patient group with high levels of ONECUT3 expression had a high risk of poor overall survival, even if accompanied by high infiltration of NK cells (Fig. 3O, Supplementary Fig. S13). Meanwhile, we utilized the datasets of PDAC from ICGC to validate the above results. Expectedly, the results were consistent with those obtained from TCGA datasets (Supplementary Fig. S14). Therefore, it can be inferred that stemness-high ONECUT3^+^ PDACs may impair the tumoricidal function of NK cells by expressing inhibitory immune checkpoints such as LGALS3, NECTIN2 and LGALS9.

### Regulation of the stemness‑related transcription factor in PDAC

**Figure 4:**
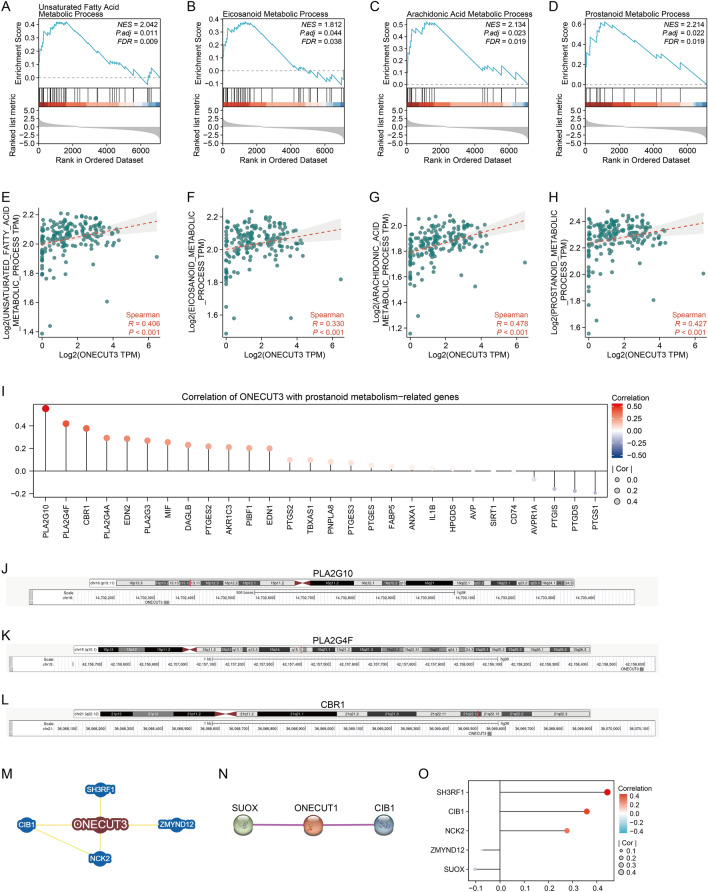
Regulation of the stemness-related transcription factor ONECUT3 in PDAC. (**A**–**D**) GSEA of differentially expressed genes between PDACs with high and low levels of ONECUT3 expression. (**E**–**H**) Spearman's correlation of ONECUT3 expression with (**E**) unsaturated fatty acid (**F**) eicosanoid, (**G**) arachidonic acid and (**H**) prostanoid metabolic process. (**I**) Spearman's correlation of ONECUT3 expression with the prostanoid metabolism-related genes. (**J**) Schematic diagram of the transcriptional regulation of PLA2G10 by ONECUT3. (**K**) Schematic diagram of the transcriptional regulation of PLA2G4F by ONECUT3. (**L**) Schematic diagram of the transcriptional regulation of CBR1 by ONECUT3. (**M**) Network of ONECUT3-interacting proteins obtained from BioGRID database. (**N**) Network of ONECUT3-interacting proteins obtained from STRING database. (**O**) Spearman's correlation of ONECUT3 expression with the five ONECUT3-interacting proteins.

To figure out the regulatory pathways of ONECUT3, we performed GSEA by comparing PDACs with high and low levels of ONECUT3 expression and characterized ONECUT3^high^ PDACs at the transcriptomic level. ONECUT3^high^ PDACs were enriched with the metabolic process of arachidonic acid and prostanoid, both of which belong to the eicosanoid class of the unsaturated fatty acid family (Fig. 4A–D). Correlation analysis also showed that these metabolic processes were significantly associated with ONECUT3 expression, as expected (Fig. 4E–H). Of note, cyclooxygenase converts arachidonic acids to prostanoids. As the pancreatic progenitor tumour subtype is characterized by gene programmes regulating fatty acid metabolism^37^, it is reasonable that the prostanoid metabolic process is abnormally activated in ONECUT3^high^ PDACs, most of which can be catalogued into pancreatic progenitor tumours. Meanwhile, considering that prostanoid metabolism hyperactivates oncogenic signalling and remodels the tumour microenvironment^40^, we wondered whether prostanoid metabolism regulates cancer stemness and immune evasion. As a result, the prostanoid metabolic process was enriched in PDACs with high mRNAsi scores (Fig. 3E) and high NK cell-targeted immune checkpoint expression (Supplementary Fig. S15), which implies that prostanoid metabolism may mediate cancer stemness and immune evasion in ONECUT3^high^ PDACs. Furthermore, to figure out whether ONECUT3 directly regulates prostanoid metabolism-related genes, we analysed the correlation of ONECUT3 with those genes and predicted the transcription factors of these genes to see if ONECUT3 is among them. Consequently, PLA2G10, PLA2G4F and CBR1, which are involved in prostanoid biosynthesis, were found to be correlated with ONECUT3 (Fig. 4I). The prediction by JASPAR CORE 2022 showed that ONECUT3 could be one of the transcription factors binding to the promoter of PLA2G10, PLA2G4F and CBR1 (Fig. 4J–L). These results suggest that ONECUT3 may mediate prostanoid metabolism by transcriptionally regulating PLA2G10, PLA2G4F and CBR1, which orchestrates cancer stemness and immune evasion.

**Figure 5:**
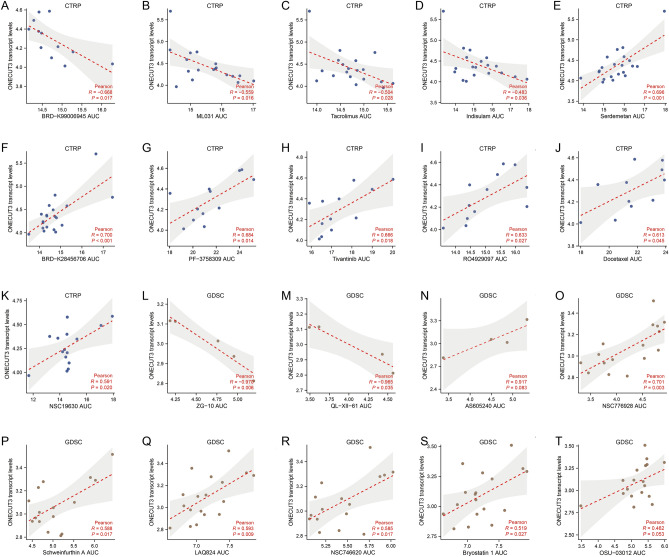
Association of ONECUT3 expression with and sensitivity to small molecular inhibitors. (**A**–**D**) Based on CTRP, Pearson’s correlation of ONECUT3 expression with drug sensitivity to (**A**) BRD-K99006945, (**B**) ML031, (**C**) tacrolimus and (**D**) indisulam. (**E**–**K**) Based on CTRP, Pearson’s correlation of ONECUT3 expression with drug sensitivity to (**E**) serdemetan, (**F**) BRD-K28456706, (**G**) PF-3758309, (**H**) tivantinib, (**I**) RO4929097, (**J**) docetaxel and (**K**) NSC19630. (**L**–**M**) Based on GDSC, Pearson’s correlation of ONECUT3 expression with drug sensitivity to (**L**) ZG-10 and (**M**) QL-XII-61. (**N**–**T**) Based on GDSC, Pearson’s correlation of ONECUT3 expression with drug sensitivity to (**N**) AS605240, (**O**) NSC776928, (**P**) Schweinfurthin, (**Q**) LAQ824, (**R**) NSC756620, (**S**) bryostatin and (**T**) OSU-03012.

Five ONECUT3-interacting proteins were obtained via experiment evidence in BioGRID and STRING database (Fig. 4M,N). We found that the expression levels of CIB1 and SH3RF1 were significantly correlated with that of ONECUT3 (Fig. 4O). SH3RF1 is a multifunctional protein containing an N-terminus RING-finger and four SH3 domains. It has E3 ubiquitin-protein ligase activity and can catalyze self-ubiquitination without an external substrate^41^. SH3RF1 can stimulate the ubiquitination and the clathrin-independent endocytosis of the potassium channel ROMK1^42^. It is also involved in the regulation of Ca^2^^+^ homeostasis by decreasing the surface levels of the endoplasmic reticulum calcium sensor STIM1^43^. As CIB1 is a positive transcriptional regulator^44^ and SH3RF1 is a negative post-transcriptional regulator^45^, CIB1 may act as a transcriptional co-activator binding with ONECUT3, and SH3RF1 may function as a negative feedback regulator interacting with ONECUT3. Furthermore, the potential drugs sensitive to ONECUT3^high^ PDACs with high stemness were screened using CTRP and GDSC. The PDAC cell lines with high levels of ONECUT3 expression produced fewer ATPs when exposed to BRD-K99006945, ML031, tacrolimus, indisulam, ZG-10 and QL-XII-61 (Fig. 5A–D, L and M), which means that these small inhibitors could efficiently target ONECUT3^high^ PDACs. Conversely, treatment with serdemetan, BRD-K28456706, PF-3758309, tivantinib, RO4929097, docetaxel, NSC19630, AS605240, NSC776928, Schweinfurthin, LAQ824, NSC746620, Bryostatin and OSU-03012 had a better impact on the PDAC cell lines with lower levels of ONECUT3 expression (Fig. 5E–K,N–T), which means that these small inhibitors could be used for clearance of ONECUT3^low/-^ PDACs. Therefore, the blockade of prostanoid metabolism and the application of those small inhibitors during precision immunotherapy may synergize with ICB to eliminate cancer stemness and immune evasion in ONECUT3^high^ PDACs.

## Discussion

Considering tumour heterogeneity, precision immunotherapy requires a comprehensive understanding of cancer stem cell immunology. In this study, we confirmed the influence of PDAC stemness on the infiltration of NK cells and identified ONECUT3 as a candidate stemness-related transcription factor that regulates NK cell-targeted inhibitory immune checkpoints in PDAC. The patient group with high levels of ONECUT3 expression had a high risk of poor overall survival, even if accompanied by high infiltration of NK cells. Moreover, ONECUT3 enriched in high-stemness PDACs possessed the potential to transcriptionally regulate the prostanoid metabolism-related genes.

The intertwined relationship between stemness and immune evasion lays the groundwork for precision immunotherapy targeting cancer stem cells. PARP1^high^ acute myeloid leukaemia, which lacked NKG2D ligands but showed functional stemness characteristics, initiated serially re-transplantable leukaemia, whereas transfer of polyclonal NK cells, after treatment with PARP1 inhibitors which restored NKG2D ligand expression, suppressed leukaemogenesis^46^. Genetic depletion and pharmacological inhibition of FTO not only dramatically attenuated self-renewal of leukaemia stem cells, but also reprogrammed immune response by suppressing LILRB4 expression^47^. In squamous cell carcinoma, tumour-initiating cells acquired the immune checkpoint molecules CD80 and CD276 to evade immunological surveillance from cytotoxic lymphocytes during local recurrence and metastasis^16,17^. Our study revealed ONECUT3 as a candidate stemness-related transcription factor positively associated with NK cell-targeted inhibitory immune checkpoints such as LGALS3, LGALS9 and NECTIN2. Transcription factor ONECUT3 regulates intrahepatic biliary development in zebrafish^48^. It initiates mucin gene transcription and activates mucin glycosylation in intrahepatic mucinous cholangiocarcinoma^49^. ONECUT3 expression was also found in the developing pancreas and, however, undetectable in the absence of ONECUT1^50^. Notably, ONECUT3 increases extracellular acidification by reprogramming glycolysis, consequently impairing CD8^+^ T cell infiltration and leading to poor response to anti-PD-1 therapy^51^. Our findings suggest that the regulation of inhibitory signals for NK cell activation by ONECUT3 may protect the rare population of cancer stem cells from robust anti-tumour immunity during distant metastasis or local recurrence. Uncovering ONECUT3^high^ PDAC with high stemness and immune privilege increases the possibility of applying NK cell-based precision immunotherapy for PDAC stem cells.

NK cells comprise a unique population of innate lymphoid cells endowed with intrinsic abilities to swiftly eliminate adjacent tumour cells. Decreased frequency and activity of NK cells in the peripheral blood of PDAC patients were correlated with unsatisfactory responses to chemotherapy^52,53^. Impaired cytotoxicity of NK cells in PDAC tissues forecasted shorter overall and progression-free survival^54^. Similarly, our study showed that patients with a lower proportion of tumour-infiltrating of NK cells had a risk of poor overall survival. These results underline the importance of exploring the NK cell platform for cancer immunotherapy, particularly in the context of intratumoral heterogeneity caused by cancer stem cells. Several advantages of NK cell biology can be utilized for NK-based precision immunotherapy: i) NK cells broadly recognize tumours with a very low mutational burden. Tumours with high mutational burden were found in about 1% of PDACs, while the majority of PDACs belong to the cancer type category with the lowest mutational burden^55^. Unlike CD8^+^ T cells that require neoantigen presentation through MHC-I, NK cells can respond to tumours that lack neoantigens, such as PDAC. ii) NK cells enhance activity against tumours that lost expression of MHC-I owing to acquired resistance mechanisms. PDAC cells displayed reduced expression of MHC-I, which was selectively targeted for lysosomal degradation by an autophagy-dependent mechanism^20^. More importantly, cancer stem cells exhibited reduced expression of surface MHC-I compared with non-stem cancer cells^56^, which suggests that it is favourable for NK cells to recognize and attack PDAC stem cells. iii) Inhibition of inhibitory signals transduced by immune checkpoints mediates NK cell function. Our results reveal that LGALS3, LGALS9 and NECTIN2, known as ligands of inhibitory receptors on the surface of NK cells, may act as stemness-related immune checkpoints regulated by ONECUT3. Blockade of inhibitory immune checkpoints on cancer stem cells facilitates mobilization of NK cell activity. The enormous potential of NK cells for precision immunotherapy for PDAC stem cells will emerge with an in-depth understanding of cancer stem cell immunology.

An accumulating amount of data has indicated that alteration in unsaturated fatty acid metabolism fulfils the energy demands and biomass generation of cancer stem cells and contributes to the activation of oncogenic signalling pathways. Prostanoids are a family of lipid mediators produced by the action of cyclooxygenase on arachidonic acids^57^. Prostaglandin E2 (PGE2) promoted colorectal cancer stem cell expansion and metastasis in preclinical models. Levels of PGE2 were found to be correlated with cancer stem cell markers including CD133, CD44, LGR5 and SOX2^58^. Mesenchymal stem cell-derived PGE2 created a cancer stem cell niche to enable tumour progression. Mechanically, PGE2 induces β-catenin nuclear localization and transactivation and thus increased ALDH^high^ cancer stem cell-enriched population^59^. Meanwhile, PGE2 actively triggers tumour immune evasion in many ways, leading to poor response of immunotherapy. COX-2-converted PGE2 induced PD-1 expression by enhancing binding of NF-κB to the PD-1 promoter via EP4-PI3K-AKT pathway in CD8^+^ T cells and macrophages^60^. PGE2 also interacted with EP2 and EP4 receptors on NK cells to block the release of IFN-γ, hindering the remodelling of the tumour microenvironment^61^. Pharmacological inhibition of COX-2 activity and PGE2 release potentiated PD-1/PD-L1 blockade immunotherapy^62^. Since our results showed that the prostanoid metabolic process enriched in ONECUT3^high^ PDAC is associated with high mRNAsi scores and NK cell-targeted inhibitory immune checkpoints and that ONECUT3 potentially regulates prostanoid metabolism-related genes, it is reasonable to infer that ONECUT3-mediated prostanoid metabolism may orchestrate cancer stemness and immune evasion. If combined with ICB, interference with prostanoid metabolism, such as COX-2 inhibitor, could not only attenuate cancer stemness but also break down the immune privilege of cancer stem cells within stemness-high PDAC.

Some limitations in the current study still need to be addressed in the future. First of all, our findings were based on bulk transcriptomic sequencing data from a public database. Despite deconvolution algorithms to analyse tumour cells and their surrounding microenvironment, a single-cell transcriptomic map of PDAC can provide much more detailed information on cancer stem cell immunology. Secondly, our results were derived from bioinformatic analysis and required further experiments to verify in patient-derived xenograft or spontaneous tumour models. Thirdly, the therapeutic effect of a combination of ICB and prostanoid metabolism inhibition on local recurrence and distant metastasis led by ONECUT3^high^ PDAC stem cells requires clinical trials to manifest. Nevertheless, identifying one stemness-related transcription factor, relevant inhibitory immune checkpoints and regulatory pathways are anticipated to promote the development of cancer stem cell immunology and precision immunotherapy.

In summary, our study reveals ONECUT3 as a candidate stemness-related transcription factor regulating NK cell-targeted inhibitory immune checkpoints in PDAC. High levels of ONECUT3 expression, even if accompanied by high infiltration of NK cells, forecast poor overall survival. ONECUT3-mediated prostanoid metabolism may regulate cancer stemness and immune evasion in PDAC. Synergistic inhibition of prostanoid metabolism may improve the efficacy of NK cell-based precision immunotherapy targeting ONECUT3^high^ PDACs with cancer stemness and immune evasion.

### Supplementary Information

Supplementary Figures.

## Data Availability

The datasets analysed during the current study are available in the TCGA, GTEx, ICGC, TISCH2, HPA, CTRP, GDSC, BioGRID and STRING. TCGA: https://xenabrowser.net/datapages/. GTEx: https://www.gtexportal.org/home/datasets. ICGC: https://dcc.icgc.org/releases/current/Projects. TISCH2: http://tisch.comp-genomics.org/gallery/. HPA: https://www.proteinatlas.org/about/download. CTRP: https://ctd2-data.nci.nih.gov/Public/Broad/CTRPv2.0_2015_ctd2_ExpandedDataset/. GDSC: https://www.cancerrxgene.org/downloads/bulk_download. BioGRID: https://downloads.thebiogrid.org/BioGRID. STRING: https://cn.string-db.org/cgi/download?sessionId=b3aGQloB0PPs.
