# Supplementary material for: Identification of ONECUT3 as a stemness-related transcription factor regulating NK cell-mediated immune evasion in pancreatic cancer
Source: Sci Rep. 2023 Oct 24;13:18133. doi: 10.1038/s41598-023-45560-y (PMC10598193; doi:10.1038/s41598-023-45560-y)
Supplement: Supplementary file 1 — Supplementary Figures. [file 41598_2023_45560_MOESM1_ESM.pdf]

Supplementary Figure

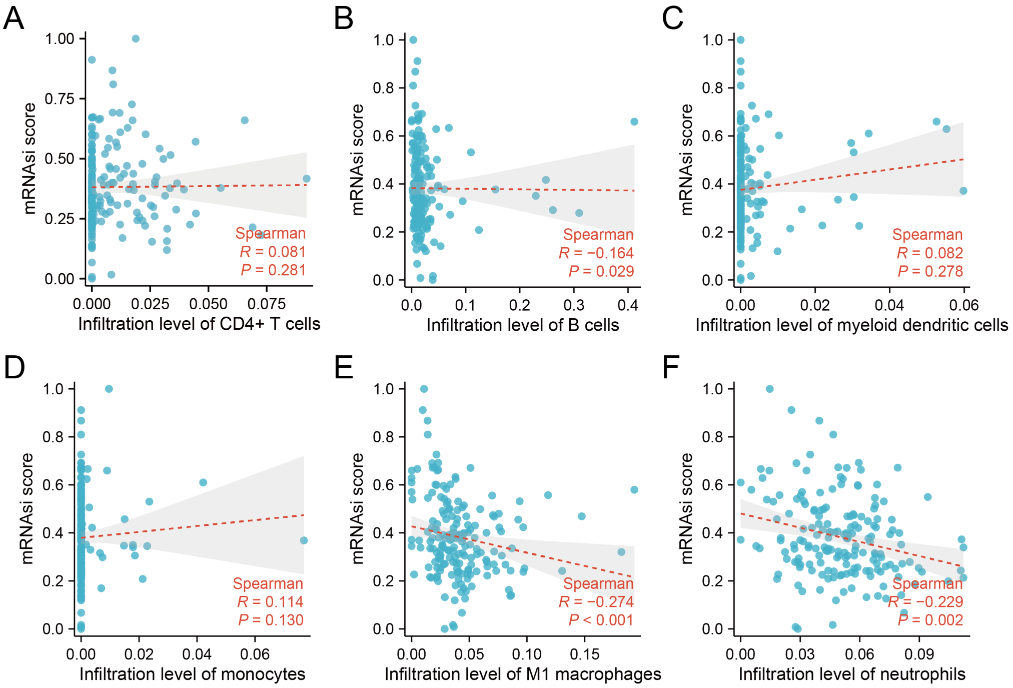

Figure S1. Association of cancer stemness with other immune infiltrates in PDAC (based on quanTIseq algorithm). (**A**) Spearman's correlation of mRNAsi scores with infiltration of $CD4^+$ T cells. (**B**) Spearman's correlation of mRNAsi scores with infiltration of B cells. (**C**) Spearman's correlation of mRNAsi scores with myeloid dendritic cells. (**D**) Spearman's correlation of mRNAsi scores with monocytes. (**E**) Spearman's correlation of mRNAsi scores with M1 macrophages. (**F**) Spearman's correlation of mRNAsi scores with neutrophils.

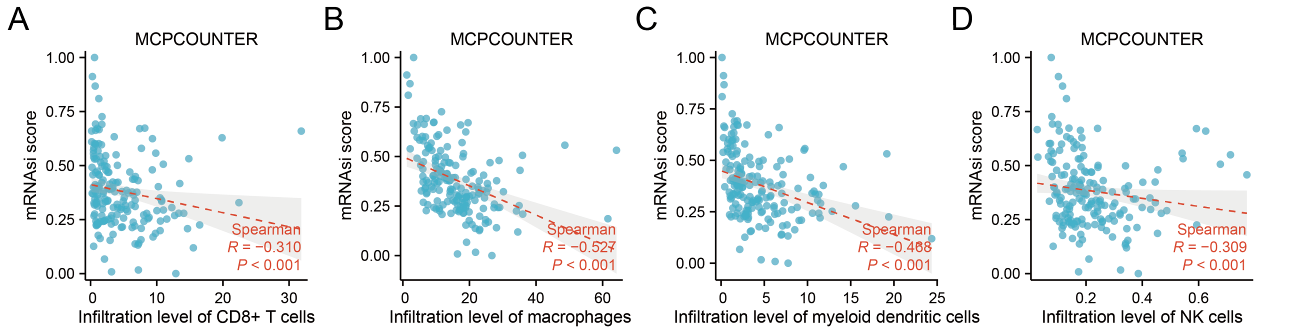

Figure S2. Association of cancer stemness with immune infiltrates in PDAC (based on MCP-counter algorithm). (**A**) Spearman's correlation of mRNAsi scores with infiltration of CD8$^+$ T cells. (**B**) Spearman's correlation of mRNAsi scores with infiltration of macrophages. (**C**) Spearman's correlation of mRNAsi scores with myeloid dendritic cells. (**D**) Spearman's correlation of mRNAsi scores with NK cells.

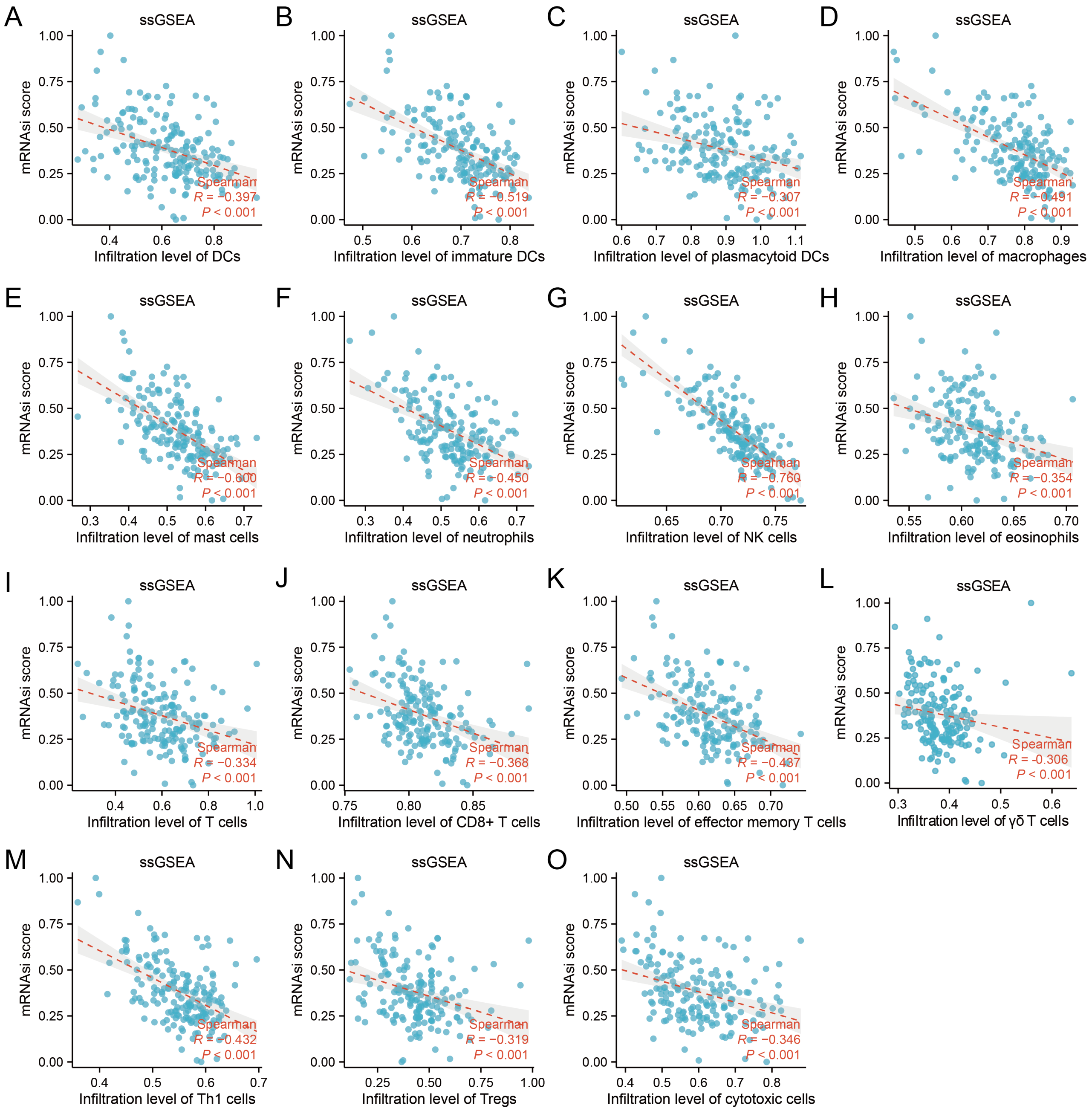

Figure S3. Association of cancer stemness with immune infiltrates in PDAC (based on ssGSEA algorithm). (**A-H**) Spearman's correlation of mRNAsi scores with infiltration of (A) dendritic cells, (B) immature dendritic cells, (C) plasmacytoid dendritic cells, (D) macrophages, (E) mast cells, (F) neutrophils, (G) NK cells and (H) eosinophils. (**I-O**) Spearman's correlation of mRNAsi scores with infiltration of (I) T cells, (J) CD8^{+} T cells, (K) effector memory T cells, (L) γδ T cells, (M) Th1 cells, (N) Tregs, (O) cytotoxic cells.

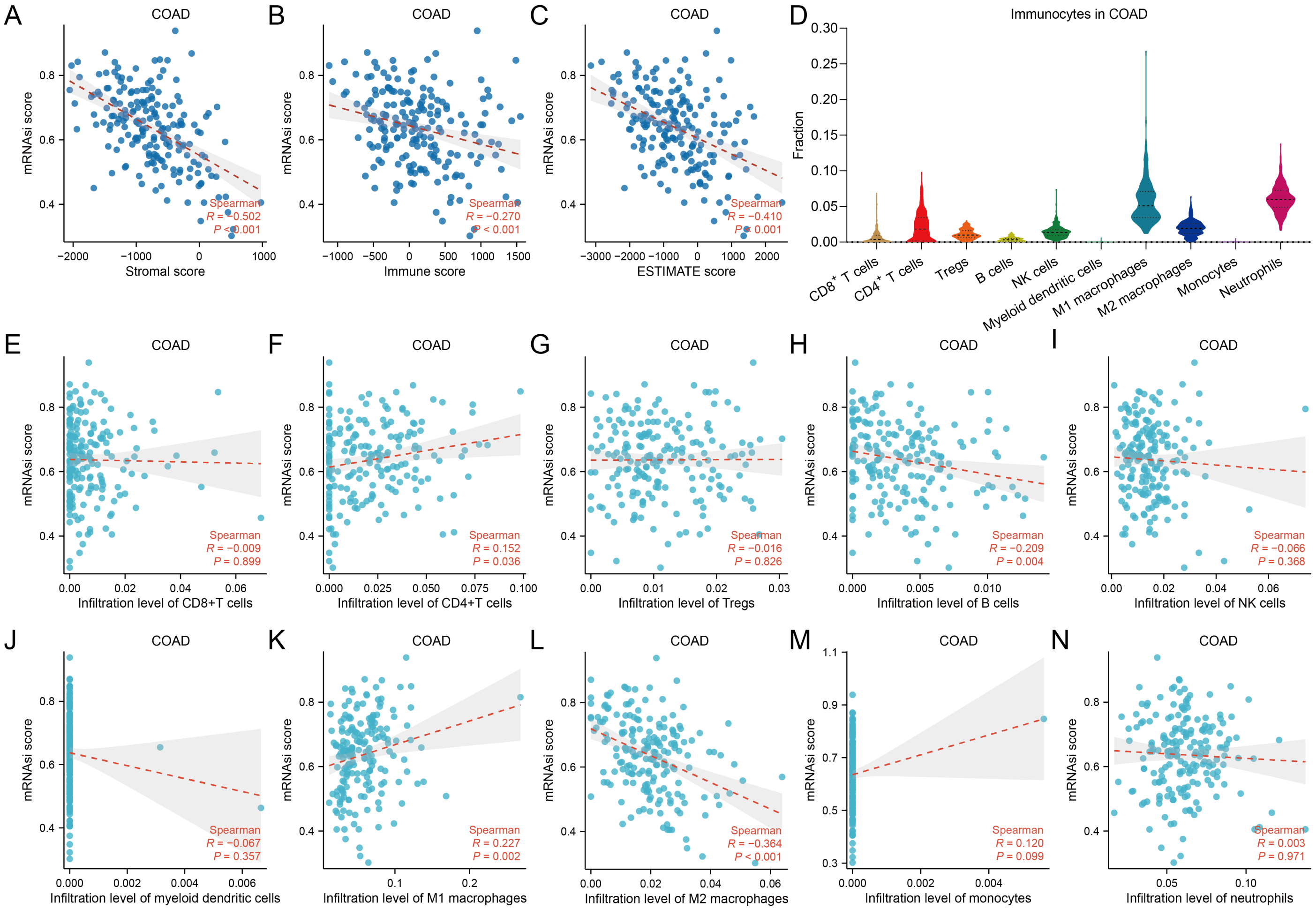

Figure S4. Association of cancer stemness with immune infiltrates in the COAD patient cohort of TCGA. (**A-C**) Spearman's correlation of mRNAsi scores with (A) Stromal scores, (B) Immune scores and (C) ESTIMATE scores in COAD (based on ESTIMATE algorithm). (**D**) The landscape of immune infiltrates in COAD (based on quanTIseq algorithm). (**E-N**) Spearman's correlation of mRNAsi scores with infiltration of (E) $CD8^+$ T cells, (F) $CD4^+$ T cells, (G) Tregs, (H) B cells, (I) NK cells, (J) myeloid dendritic cells, (K) M1 macrophages, (L) M2 macrophages, (M) monocytes and (N) neutrophils in COAD (based on quanTIseq algorithm).

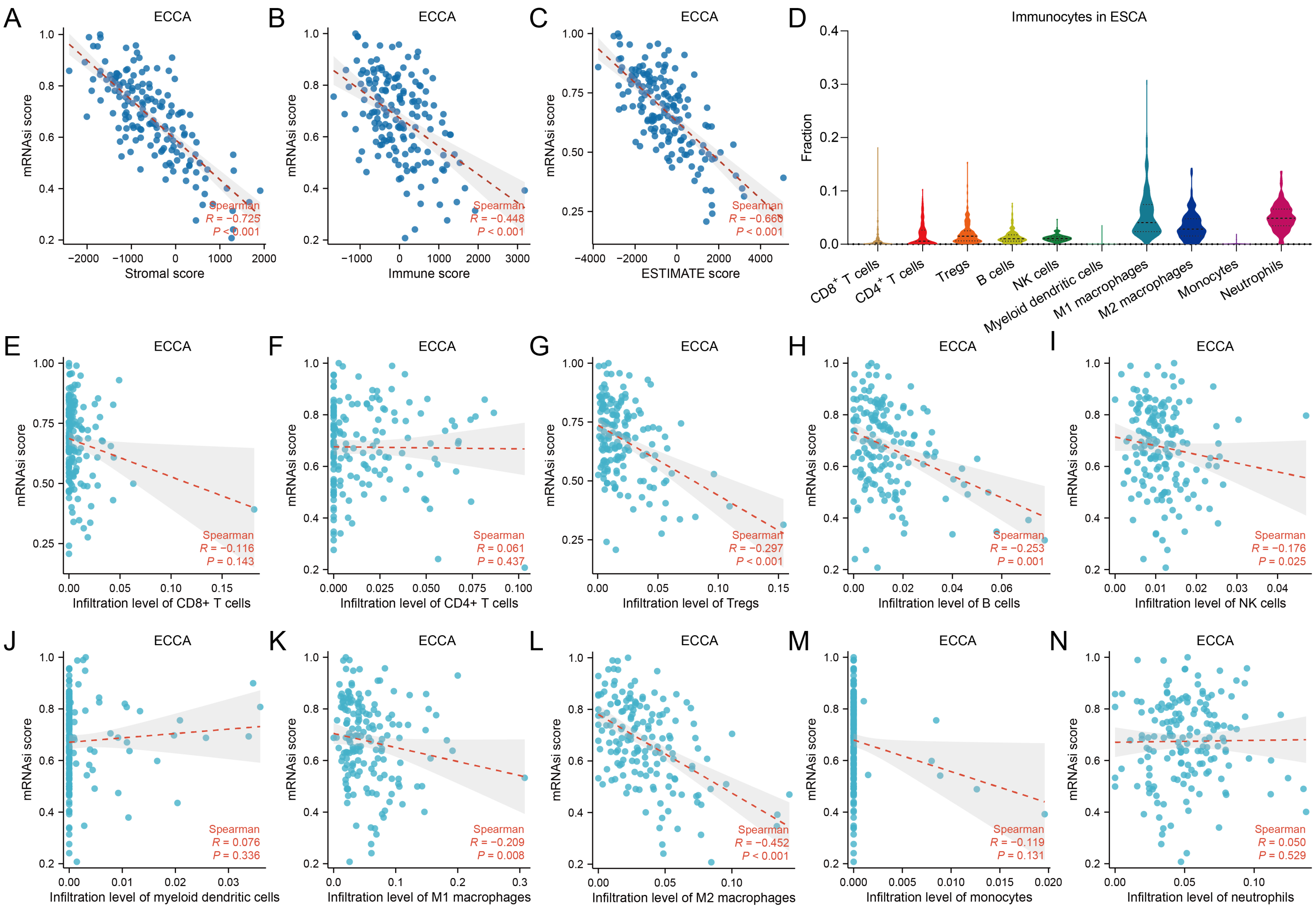

Figure S5. Association of cancer stemness with immune infiltrates in the ESCA patient cohort of TCGA. (**A-C**) Spearman's correlation of mRNAsi scores with (A) Stromal scores, (B) Immune scores and (C) ESTIMATE scores in ESCA (based on ESTIMATE algorithm). (**D**) The landscape of immune infiltrates in ESCA (based on quanTIseq algorithm). (**E-N**) Spearman's correlation of mRNAsi scores with infiltration of (E) $CD8^+$ T cells, (F) $CD4^+$ T cells, (G) Tregs, (H) B cells, (I) NK cells, (J) myeloid dendritic cells, (K) M1 macrophages, (L) M2 macrophages, (M) monocytes and (N) neutrophils in ESCA (based on quanTIseq algorithm).

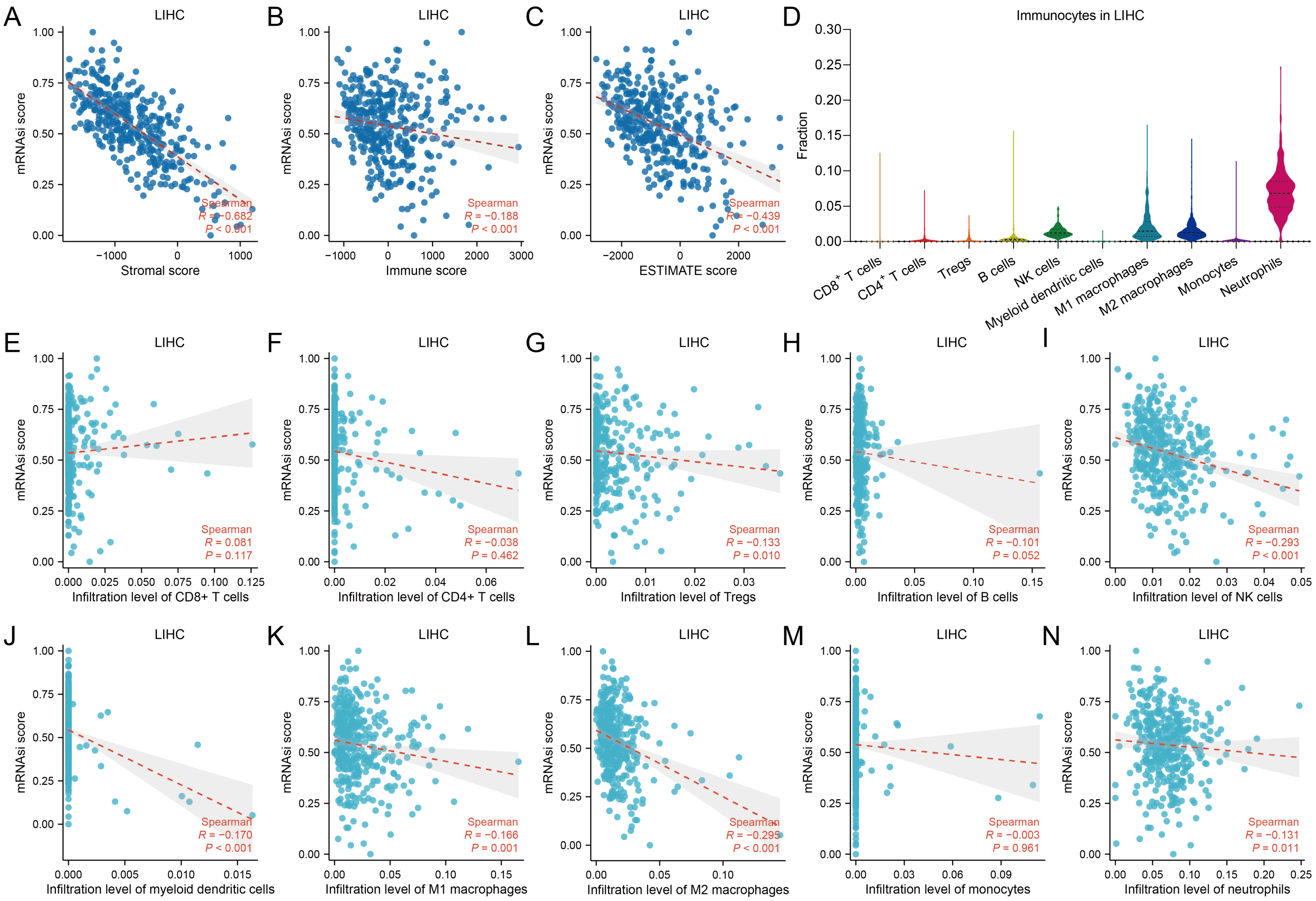

Figure S6. Association of cancer stemness with immune infiltrates in the LIHC patient cohort of TCGA. (**A-C**) Spearman's correlation of mRNAsi scores with (A) Stromal scores, (B) Immune scores and (C) ESTIMATE scores in LIHC (based on ESTIMATE algorithm). (**D**) The landscape of immune infiltrates in LIHC (based on quanTIseq algorithm). (**E-N**) Spearman's correlation of mRNAsi scores with infiltration of (E) $CD8^+$ T cells, (F) $CD4^+$ T cells, (G) Tregs, (H) B cells, (I) NK cells, (J) myeloid dendritic cells, (K) M1 macrophages, (L) M2 macrophages, (M) monocytes and (N) neutrophils in LIHC (based on quanTIseq algorithm).

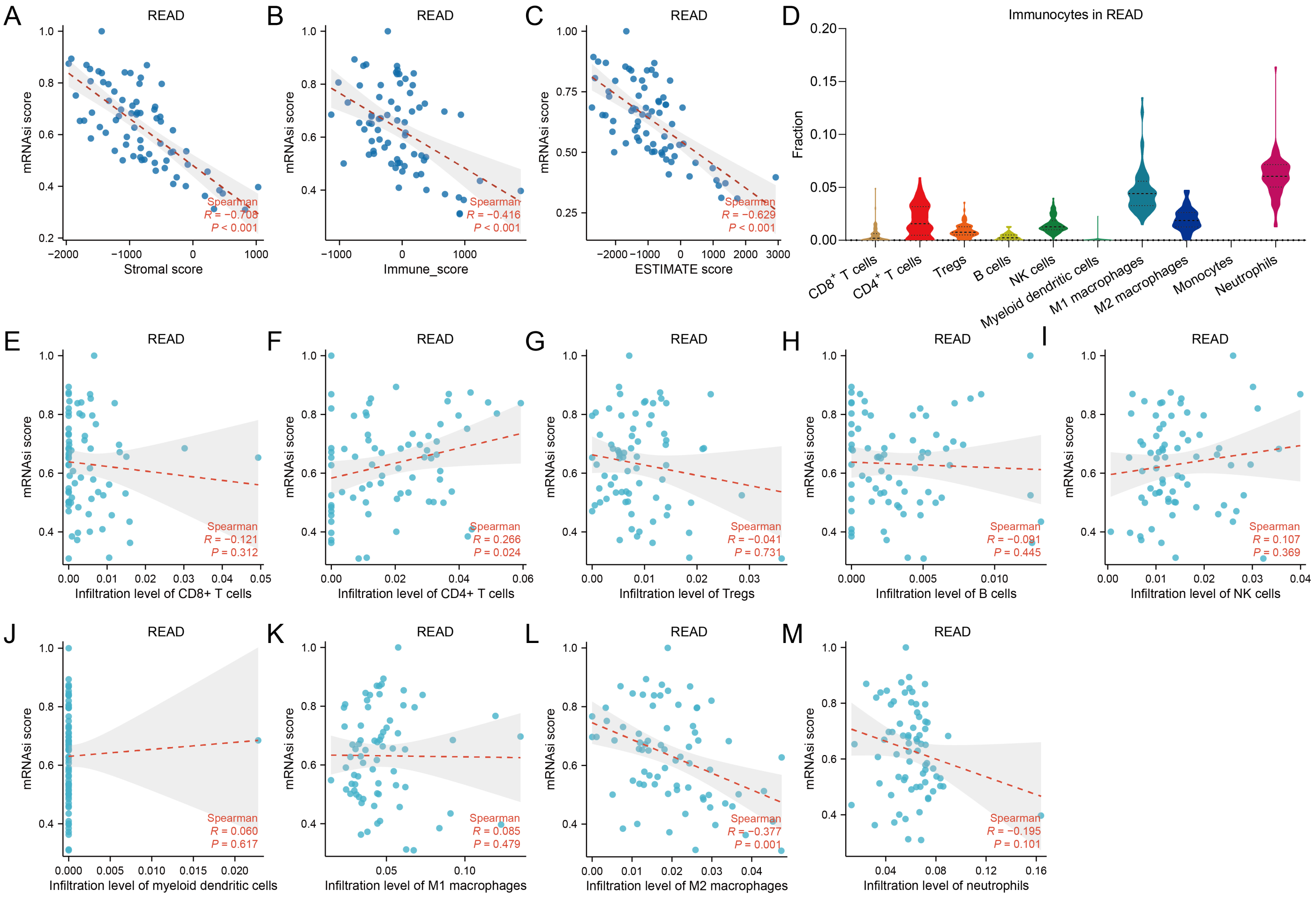

Figure S7. Association of cancer stemness with immune infiltrates in the READ patient cohort of TCGA. (**A-C**) Spearman's correlation of mRNAsi scores with (A) Stromal scores, (B) Immune scores and (C) ESTIMATE scores in READ (based on ESTIMATE algorithm). (**D**) The landscape of immune infiltrates in READ (based on quanTIseq algorithm). (**E-M**) Spearman's correlation of mRNAsi scores with infiltration of (E) CD8^{+} T cells, (F) CD4^{+} T cells, (G) Tregs, (H) B cells, (I) NK cells, (J) myeloid dendritic cells, (K) M1 macrophages, (L) M2 macrophages and (M) monocytes in READ (based on quanTIseq algorithm).

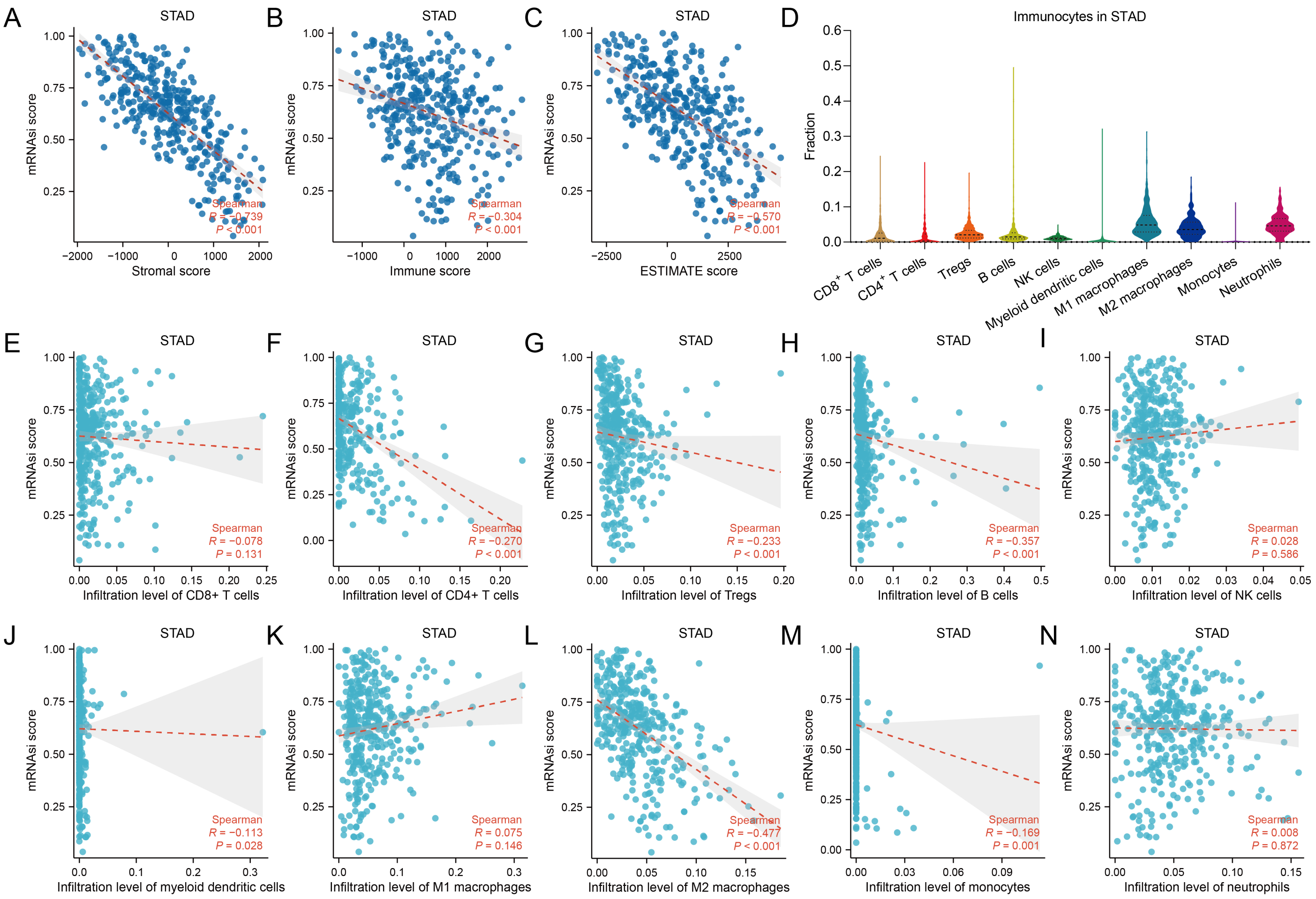

Figure S8. Association of cancer stemness with immune infiltrates in the STAD patient cohort of TCGA. (**A-C**) Spearman's correlation of mRNAsi scores with (A) Stromal scores, (B) Immune scores and (C) ESTIMATE scores in STAD (based on ESTIMATE algorithm). (**D**) The landscape of immune infiltrates in STAD (based on quanTIseq algorithm). (**E-N**) Spearman's correlation of mRNAsi scores with infiltration of (E) $CD8^+$ T cells, (F) $CD4^+$ T cells, (G) Tregs, (H) B cells, (I) NK cells, (J) myeloid dendritic cells, (K) M1 macrophages, (L) M2 macrophages, (M) monocytes and (N) neutrophils in STAD (based on quanTIseq algorithm).

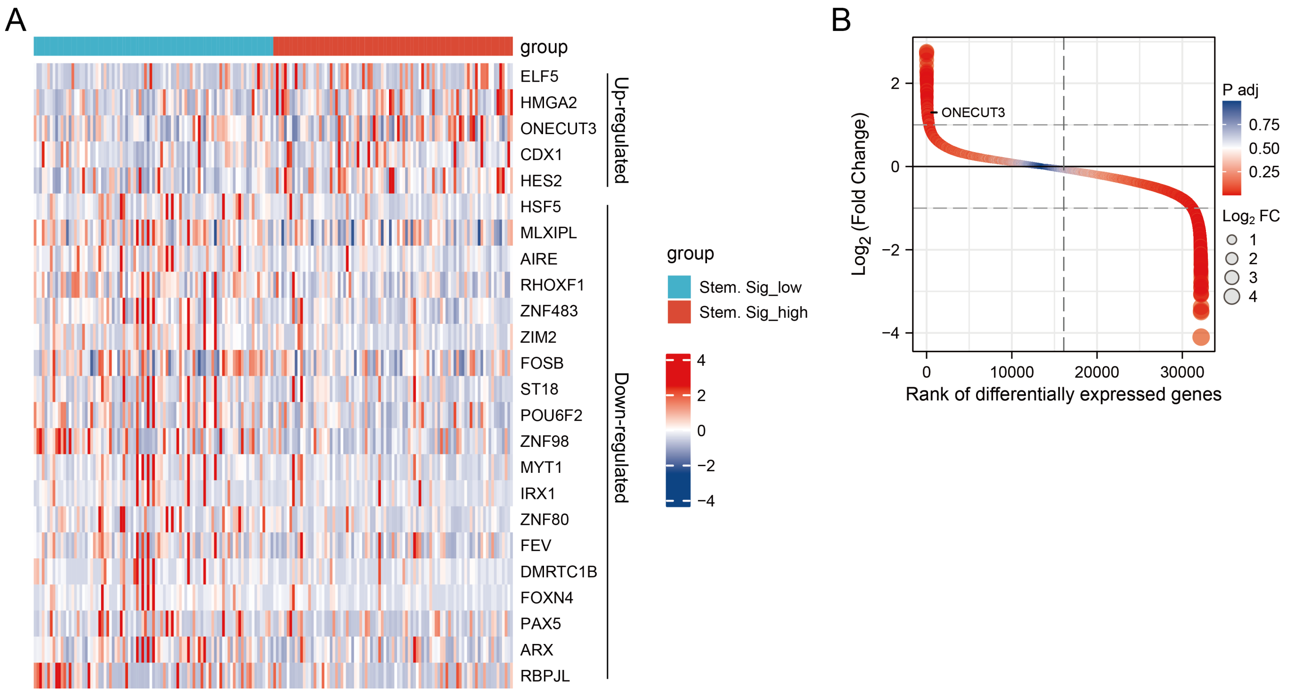

Figure S9. Identification of stemness-related transcription factors in PDACs based on Pan-cancer Stemness Signature (PSS). (**A**) Heatmap of differentially expressed transcription factors in PDACs with low and high PSS levels. (**B**) The rank of differentially expressed transcription factors according to log2(Fold Change).

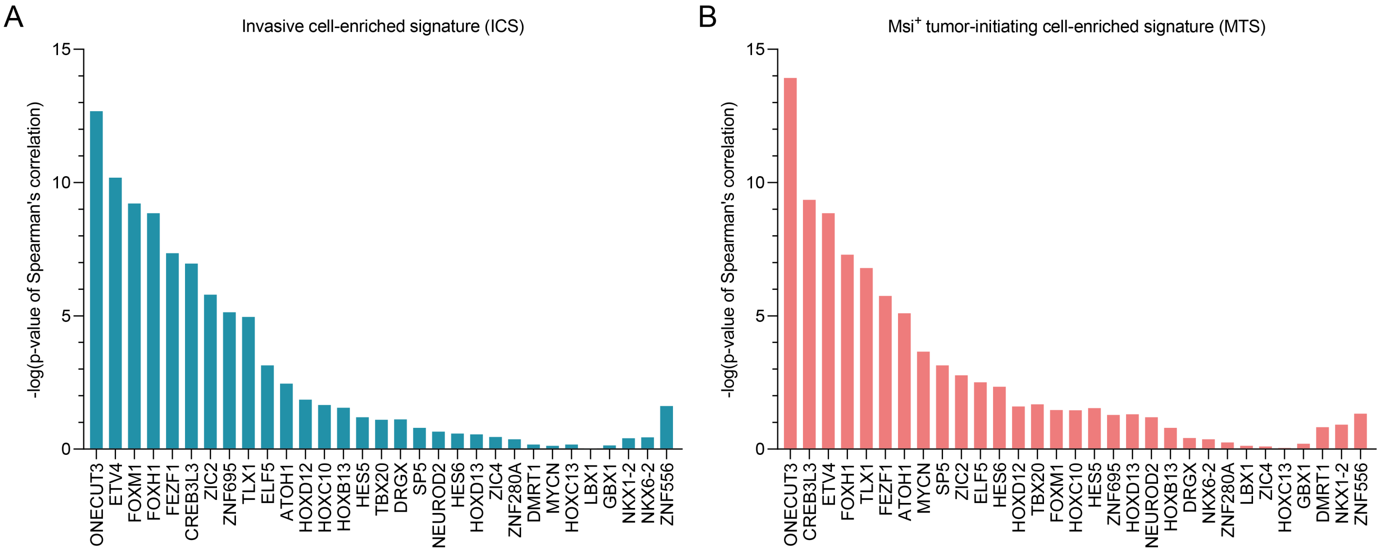

Figure S10. Statistical significance of association between the transcription factors and two individual sets of gene signatures in PDAC. (**A**) The *p*-value of Spearman's correlation between the transcription factors and CRS. (**B**) The *p*-value of Spearman's correlation between the transcription factors and MTS.

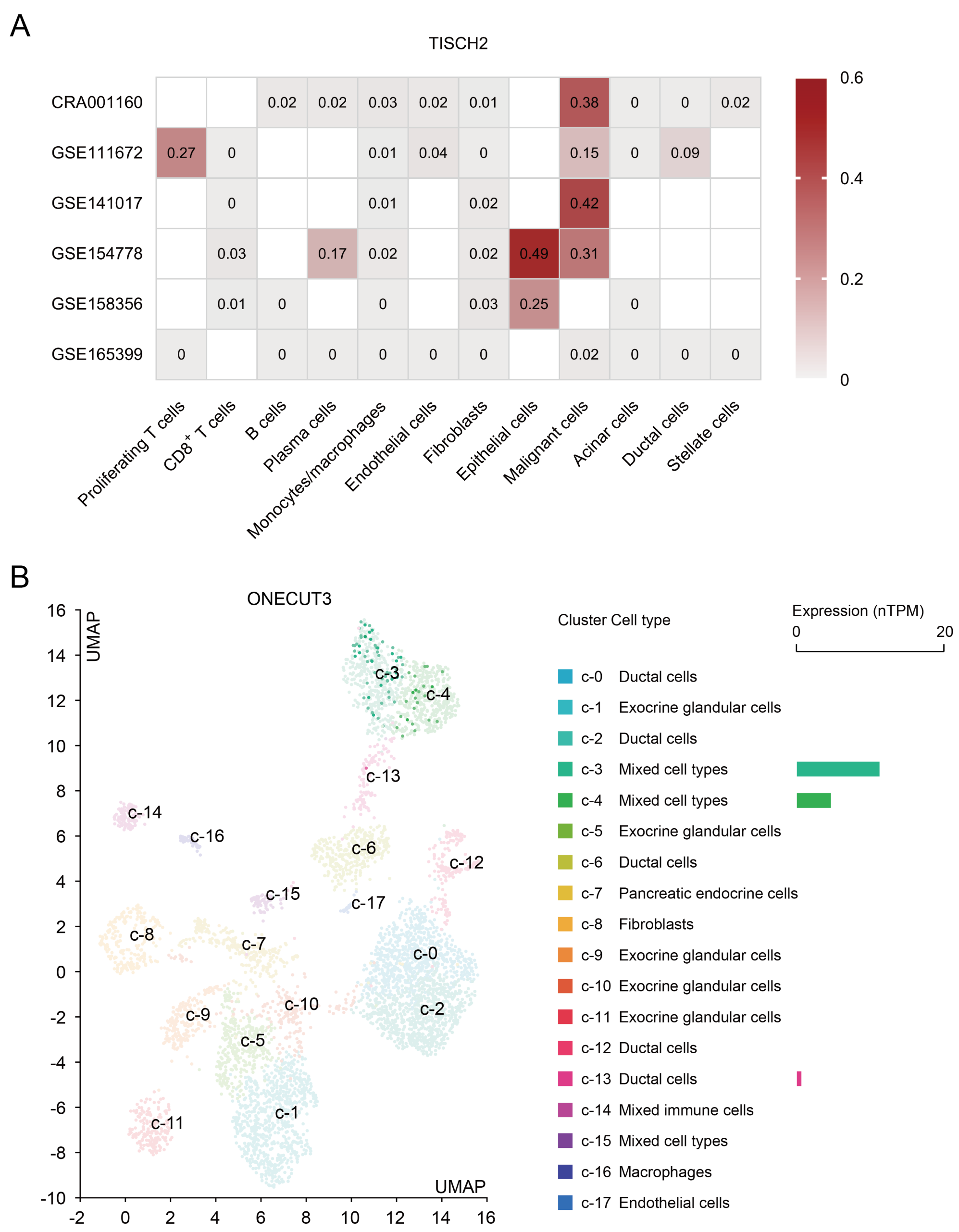

Figure S11. Single-cell transcriptomic analysis of PDAC and normal pancreas. (**A**) Heatmap of ONECUT3 expression in the CRA001160, GSE111672, GSE141017, GSE154778, GSE158365 and GSE165399 single-cell data collected from TISCH2. (**B**) UMAP plot of ONECUT3 expression in the single-cell analysis of normal pancreas derived from the HPA.

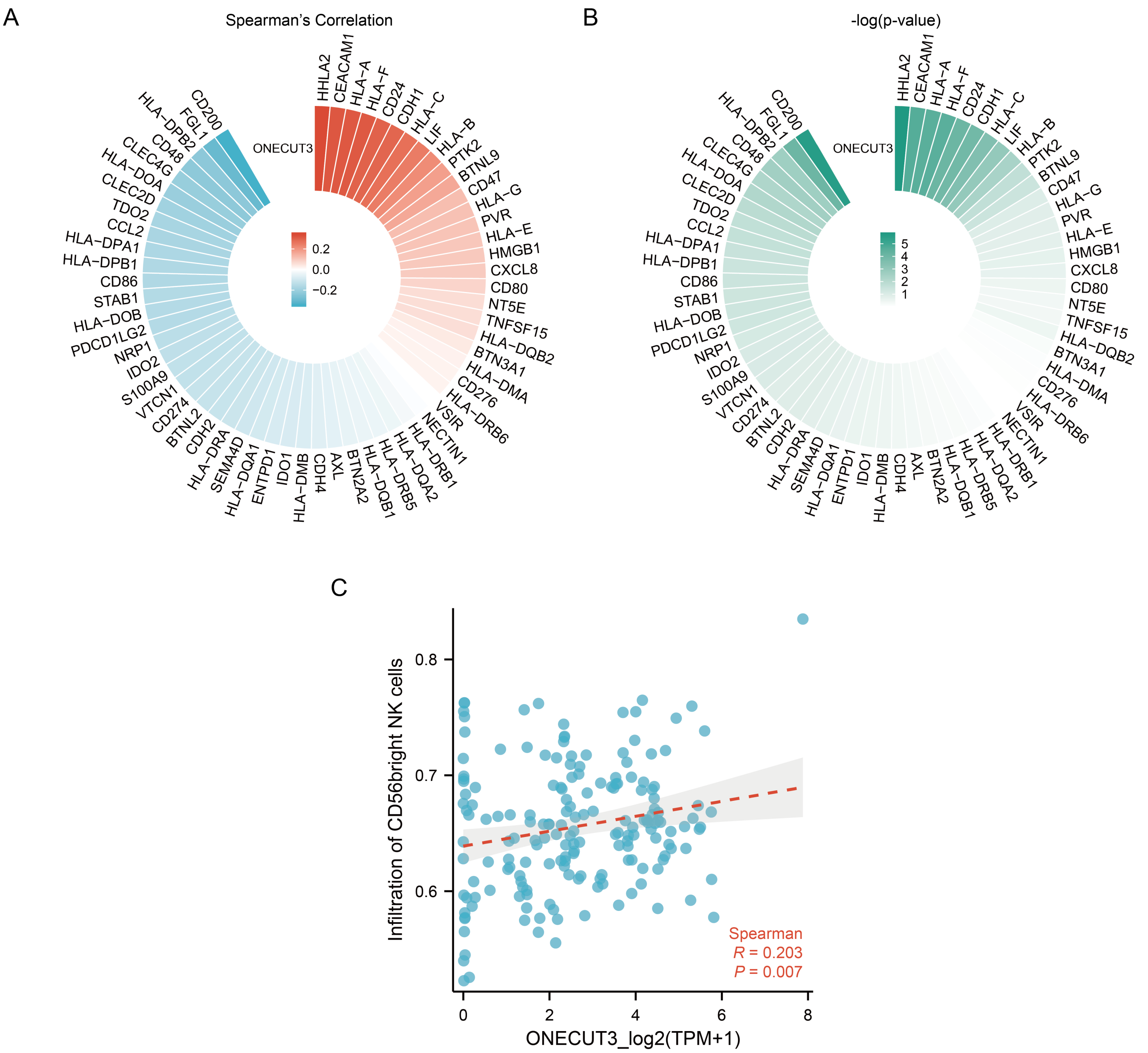

Figure S12. Influence of the stemness-related transcription factor ONECUT3 on the immune microenvironment in PDAC. (**A**) Spearman's correlation of ONECUT3 expression with the other inhibitory immune checkpoints. (**B**) The *p*-value of Spearman's correlation between ONECUT3 expression and the other inhibitory immune checkpoints. (**C**) Spearman's correlation of ONECUT3 expression with infiltration of CD56^{bright} NK cells.

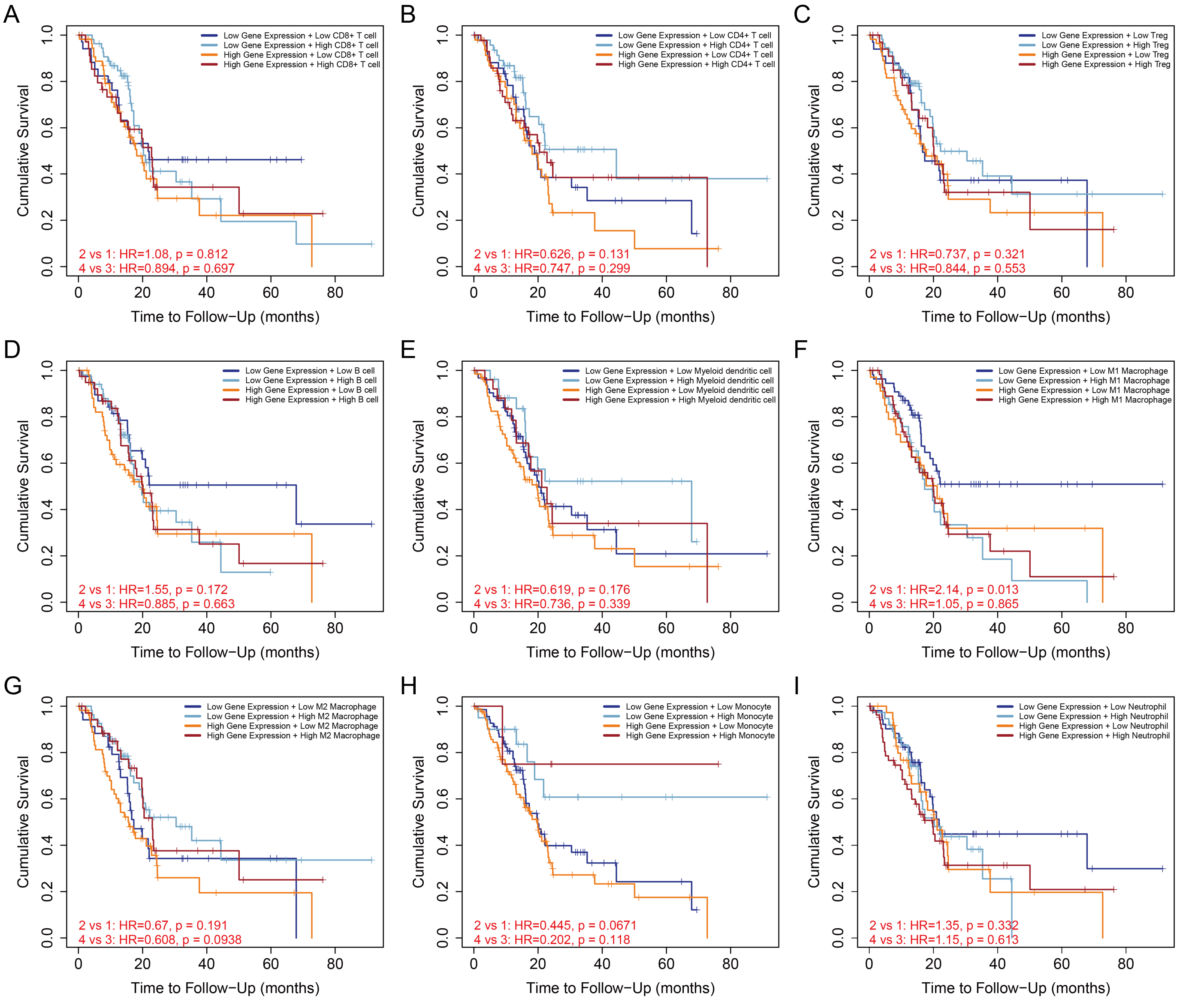

Figure S13. Prognostic value of ONECUT3 expression plus immune infiltration level in PDAC patients. (**A-D**) Kaplan-Meier curves for overall survival split by level of ONECUT3 expression and infiltration level of (A) $CD8^+$ T cells, (B) $CD4^+$ cells, (C) Tregs and (D) B cells. (**E-I**) Kaplan-Meier curves for overall survival split by level of ONECUT3 expression and infiltration level of (E) myeloid dendritic cells, (F) M1 macrophages, (G) M2 macrophages, (H) monocytes and (I) neutrophils.

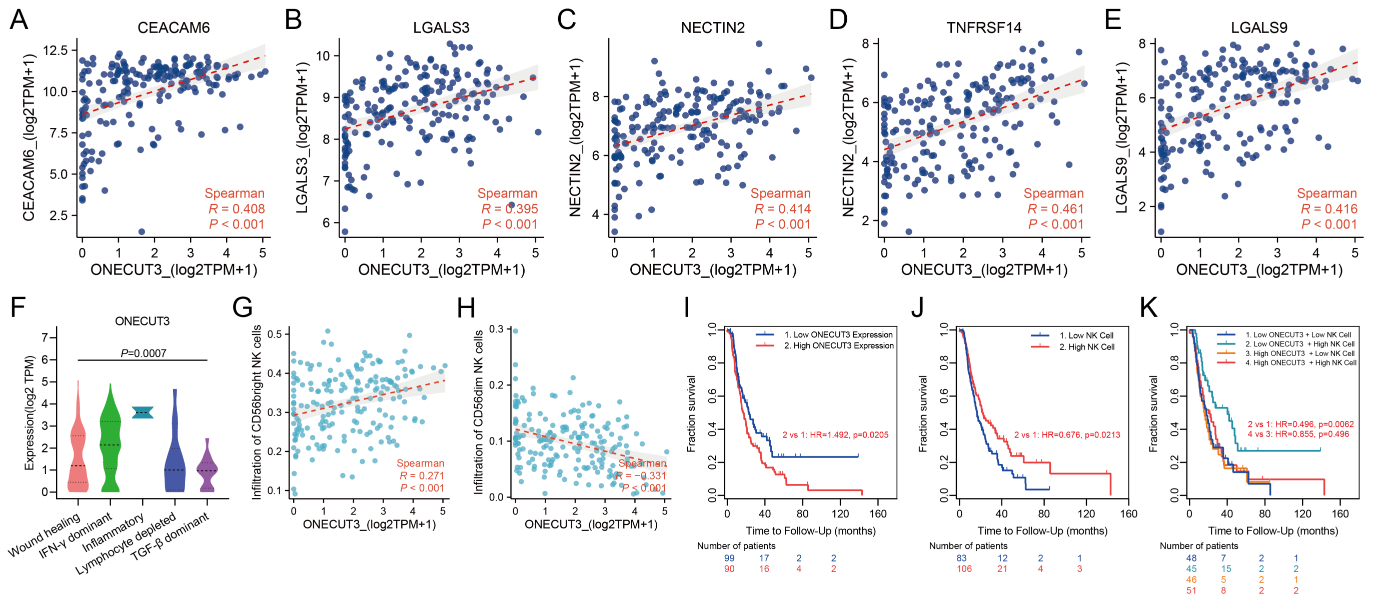

Figure S14. Validation of the prognostic value of ONECUT3 in the PDAC patient cohort from ICGC. (**A-E**) Spearman's correlation of ONECUT3 expression with (A) CEACAM6, (B) LGALS3, (C) NECTIN2, (D) TNFRSF14 and (E) LGALS9 expression in PDAC. (**F**) Violin plots for ONECUT3 expression in multiple immune subtypes of PDAC. (**G**) Spearman's correlation between ONECUT3 expression and infiltration of CD56^{bright} NK cells. (**G**) Spearman's correlation between ONECUT3 expression and infiltration of CD56^{dim} NK cells. (**I**) Kaplan-Meier curves for overall survival split by level of ONECUT3 expression in PDAC. (**J**) Kaplan-Meier curves for overall survival split by infiltration level of NK cells in PDAC. (**K**) Kaplan-Meier curves for overall survival split by level of ONECUT3 expression and infiltration level of NK cells in PDAC.

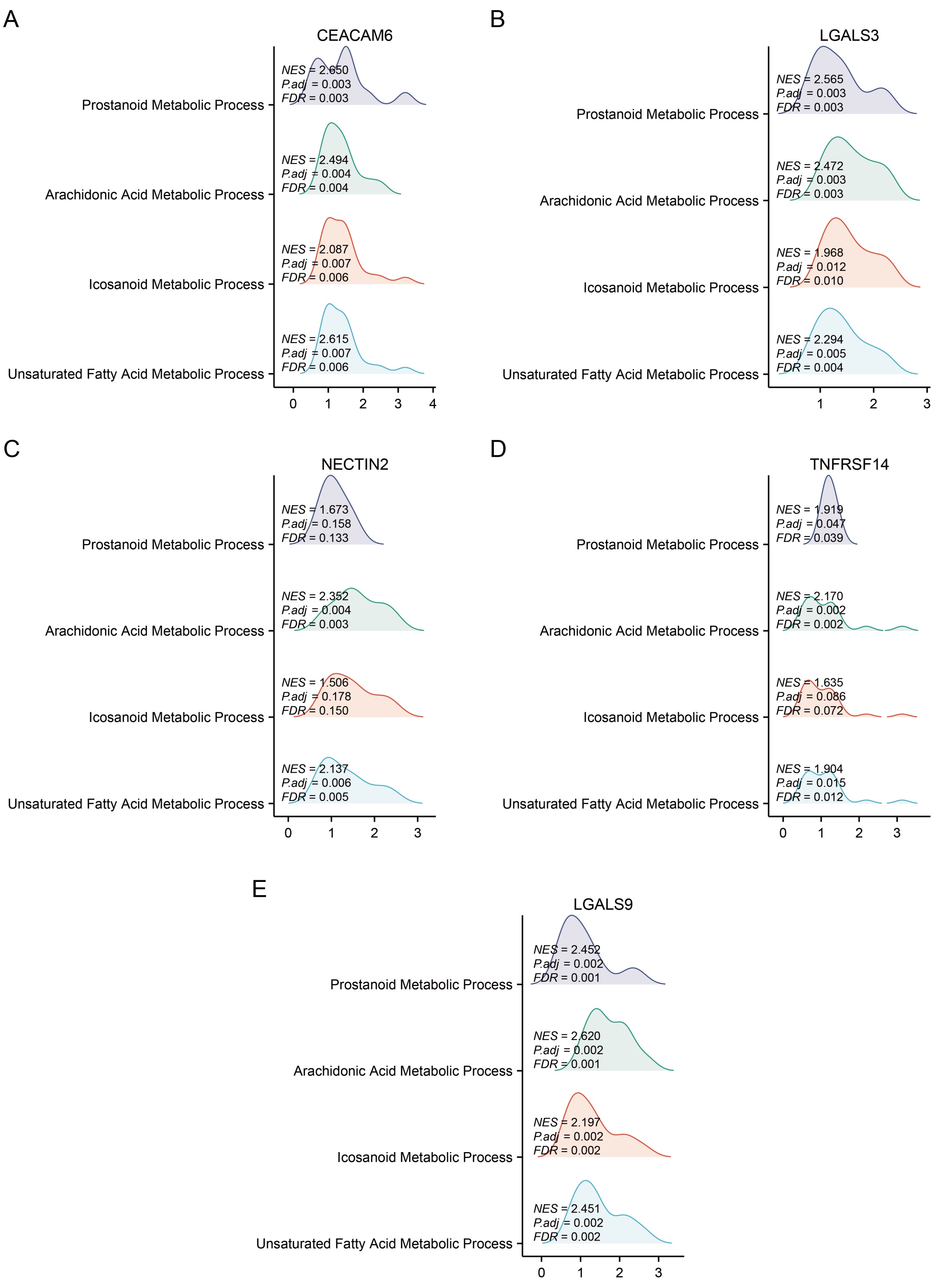

Figure S15. Metabolic processes associated with NK cell-targeted inhibitory immune checkpoints in PDAC. (**A**) Ridgeline plot for GSEA of differentially expressed genes between PDACs with high and low CEACAM6 expression. (**B**) Ridgeline plot for GSEA of differentially expressed genes between PDACs with high and low LGALS3 expression. (**C**) Ridgeline plot for GSEA of differentially expressed genes between PDACs with high and low NECTIN2 expression. (**D**) Ridgeline plot for GSEA of differentially expressed genes between PDACs with high and low TNFRSF14 expression. (**E**) Ridgeline plot for GSEA of differentially expressed genes between PDACs with high and low LGALS9 expression.
